# Long-term glucocorticoid treatment and high relapse rate remain unresolved issues in the real-life management of polymyalgia rheumatica: a systematic literature review and meta-analysis

**DOI:** 10.1007/s10067-021-05819-z

**Published:** 2021-08-20

**Authors:** Alberto Floris, Matteo Piga, Elisabetta Chessa, Mattia Congia, Gian Luca Erre, Maria Maddalena Angioni, Alessandro Mathieu, Alberto Cauli

**Affiliations:** 1 Rheumatology Unit, Azienda Ospedaliero-Universitaria di Cagliari, SS554, 09042 Monserrato, Cagliari Italy; 2 Dipartimento Di Scienze Mediche E Sanità Pubblica, Università Di Cagliari, SS554, 09042 Monserrato, Cagliari Italy; 3 Rheumatology Unit, University of Sassari and AOU University Clinic of Sassari, Sassari, Italy

**Keywords:** Glucocorticoids, Meta-analysis, Observational study, Polymyalgia rheumatica, Relapse

## Abstract

A systematic review and meta-analysis were conducted, according to the PRISMA methodology, to summarize current evidence on the prevalence and predictors of long-term glucocorticoid (GC) treatment and disease relapses in the real-life management of polymyalgia rheumatica (PMR).

Out of 5442 retrieved studies, 21 were eligible for meta-analysis and 24 for qualitative analysis. The pooled proportions of patients still taking GCs at 1, 2, and 5 years were respectively 77% (95%CI 71–83%), 51% (95%CI 41–61%), and 25% (95CI% 15–36%). No significant difference was recorded by distinguishing study cohorts recruited before and after the issue of the international recommendations in 2010. The pooled proportion of patients experiencing at least one relapse at 1 year from treatment initiation was 43% (95%CI 29–56%). Female gender, acute-phase reactants levels, peripheral arthritis, starting GCs dosage, and tapering speed were the most frequently investigated potential predictors of prolonged GC treatment and relapse, but with inconsistent results. Only a few studies and with conflicting results evaluated the potential role of early treatment with methotrexate in reducing the GC exposure and the risk of relapse in PMR.

This study showed that a high rate of prolonged GC treatment is still recorded in the management of PMR. The relapse rate, even remarkable, can only partially explain the long-term GC treatment, suggesting that other and not yet identified factors may be involved. Additional research is needed to profile patients with a higher risk of long-term GC treatment and relapse and identify more effective steroid-sparing strategies.

**Table:** 

**Key Points:** *• High rate of long-term glucocorticoid (GC) treatment is recorded in polymyalgia rheumatica (PMR), being 77%, 51%, and 25% of patients still on GCs after respectively 1, 2, and 5 years.* *• A pooled relapse rate of 43% at 1 year, even remarkable, can only partially explain the long-term GC treatment in PMR.* *• Several studies have attempted to identify potential predictors of prolonged treatment with GCs and relapse, but with inconsistent results.* *• Additional research is needed to profile patients with a higher risk of long-term GC treatment and relapse and identify more effective steroid-sparing strategies.*

**Key Points:**

*• High rate of long-term glucocorticoid (GC) treatment is recorded in polymyalgia rheumatica (PMR), being 77%, 51%, and 25% of patients still on GCs after respectively 1, 2, and 5 years.*

*• A pooled relapse rate of 43% at 1 year, even remarkable, can only partially explain the long-term GC treatment in PMR.*

*• Several studies have attempted to identify potential predictors of prolonged treatment with GCs and relapse, but with inconsistent results.*

*• Additional research is needed to profile patients with a higher risk of long-term GC treatment and relapse and identify more effective steroid-sparing strategies.*

## Introduction

Polymyalgia rheumatica (PMR) is an inflammatory disease of unknown aetiology affecting people over 50. With a lifetime risk of 2.4% for women and 1.7% for men, it is one of the most widespread inflammatory rheumatic diseases of the elderly, especially in Western countries [1]. PMR is clinically characterized by severe pain and stiffness in the shoulders and pelvic girdle, constitutional symptoms, elevation of acute-phase reactants (APRs), and rapid response to glucocorticoids (GCs) [2].

Since the 1960s, when low-moderate dosages of GCs were demonstrated to rapidly correct the clinical and laboratory disease manifestations, steroids represent the cornerstone for treatment of PMR patients [3]. However, the long-term use of GCs raises significant concerns, especially in the older ones, being strongly related to well-recognized adverse effects. It was estimated that up to 43% of PMR patients experience at least one GC-related adverse event after a mean treatment duration of 31 (± 22) months, including osteoporosis, fragility fractures, arterial hypertension, diabetes mellitus, cataract, glaucoma, infections, for myocardial infarction [4].

The British Society of Rheumatology (BSR) in 2010 [5] and the European League Against Rheumatism (EULAR) with the American College of Rheumatology (ACR) in 2015 [6] endorsed recommendations for the management of PMR. These stated that treatment with GCs should be started at the dose of 12.5–25 mg of prednisone (PDN), or equivalents, and then progressively tapered up to the definite withdrawal. Thus, even in the less desirable scenario where the slowest speed for GCs tapering is applied, the end of steroid treatment should be expected by the 12th month, if no relapse occurs, or around the 15th month, if one flare occurs [6].

However, there is a widely held perception that a significant gap exists between theory and daily clinical practice, where a large proportion of patients experiences relapses and prolonged treatment with steroids, resulting in increased exposure to the risk of GC-related adverse effects [7]. Thus, accurate and reliable data on these potential critical issues in PMR management are needed to set the research agenda and possibly update the current recommendations.

This systematic review and meta-analysis aim to summarize current evidence on the prevalence and predictors of long-term treatment with GCs and relapses in the real-life management of PMR.

## Methods

### Search strategy

This work was conducted according to the PRISMA statements [8].

We searched for published studies in the English language indexed in PubMed, Scopus, Cochrane Library, and CINAHL from inception to November 2020. As it can be assumed that, if not otherwise stated, all patients with PMR are treated with GCs, the search strategy consisted of keywords referred to the study population. No further terms or exclusion criteria were included in order to identify the largest number of publications. The following keywords in combination with Medical Subject Headings (MeSH) terms and text words were applied for searching in PubMed: (“Polymyalgia Rheumatica [Mesh]) OR (polymyalgia rheumatic* [all]) OR (rheumatic polymyalg* [all]) OR (polymyalg* [all]). The same search strategy was applied for studies indexed in the other databases. We also manually screened reference lists of selected retrieved articles to identify further papers that may have been missed in the database search.

### Studies selection

The PICOS method was used to screen studies:

- Problem/population: patients affected by PMR
- Intervention: GC treatment
- Comparison: control group was not required
- Outcome: GC persistence/withdrawal and annual flare rate at 1, 2, 3, 4, and 5 years since treatment initiation for meta-analysis; GC persistence/withdrawal and flare rate over the entire follow-up for qualitative analysis
- Study design: observational prospective and retrospective longitudinal studies

Randomized clinical trials were excluded from the meta-analysis and qualitative analysis because they are characterized by pre-set GC tapering protocols and in contrast with the aim of the study do not mirror the clinical practice in a real-life setting. Publications that included in their study cohort also patients meeting criteria for giant cell arteritis (GCA) or rheumatoid arthritis (RA) were excluded.

Title, abstract, and the full report of articles identified by the search strategy were systematically and independently screened by two authors (AF and MP) regarding eligibility and exclusion criteria. The first selection was based on titles and abstracts. Full reports of articles selected in this phase were then evaluated for inclusion in the meta-analysis and/or qualitative analysis.

Disagreements regarding the selection of an article were discussed between both reviewers until consensus was reached.

### Data collection process

AF and MP independently carried out the data extraction and collected data into an electronic sheet. The former extracted data from the included studies, and the second author checked the extracted data. Disagreements were resolved by discussion between the two review authors. The following data were recorded from the included studies: first author’s name, publication year, country, type of study (i.e. prospective, retrospective), type of referral centre (i.e. rheumatology or not), population size, overall follow-up duration, mean starting GC dosage, investigated predictors of GC duration and relapses, and rate of persistence on GCs and rate of relapse at different time points (1–5 years for meta-analysis, entire follow-up for qualitative analysis). As there is not a unique and validated definition of relapse, definitions provided in the reviewed studies were categorized by recording which of the following criteria were taken into account: clinical (reappearance or worsening of symptoms), laboratory (increased APRs), and therapeutic (required increase of therapy). Furthermore, most of the studies defined relapses as any reappearance or worsening of disease activity, regardless it occurred on stable steroids dosage, during tapering, or after withdrawal. On the contrary, some studies defined relapses as a flare that occurred during steroids treatment, and recurrence as flares that occurred after their successful withdrawal. In the present study, unless otherwise specified, relapses were conventionally considered as any disease flare of disease activity, regardless of its correlation with the GC treatment. The quality of the selected studies was assessed using an adapted version of the Joanna Briggs Institute (JBI) Critical Appraisal Checklist for observational/case series studies [9, 10].

### Statistical analysis

Meta-analysis was performed to assess the proportion of patients still taking GCs at 1, 2, 3, 4, and 5 years after treatment initiation. Furthermore, a meta-analysis was performed to assess the prevalence of patients experiencing at least one disease relapse/recurrence at 1 and 2 years. The analyses were performed only when at least three studies had comparable outcomes. As we expected high heterogeneity across the selected publications, we implemented a random-effects model meta-analysis using the generic inverse variance method to calculate the pooled rate of persistence on GCs and relapse. The 95% confidence intervals (95%CIs) were calculated by the Hartung‐Knapp‐Sidik‐Jonkman method [11]. The result of every analysis was presented in forest plots. Furthermore, the effect was plotted as the inverse of the standard error to identify the risk of publication bias by visually assessing the funnel plots’ symmetry. Statistical significance was checked using Egger’s test [12]. Heterogeneity was tested using I^2^ [13]. The recruitment period (before and after 2010, when the BSR recommendation was published) and study design (retrospective and prospective) were used as criteria for sub-group analyses on the rate of prolonged GC treatment. Furthermore, a meta-regression analysis was planned to assess the contribution to the heterogeneity of the publication year, the proportion of patients on MTX, and the different definitions of relapse. Meta-essential (Version 1.5) was used for all statistical analyses [14]. A P-value < 0.05 was considered to be significant.

## Results

### Study selection

**Fig. 1:**
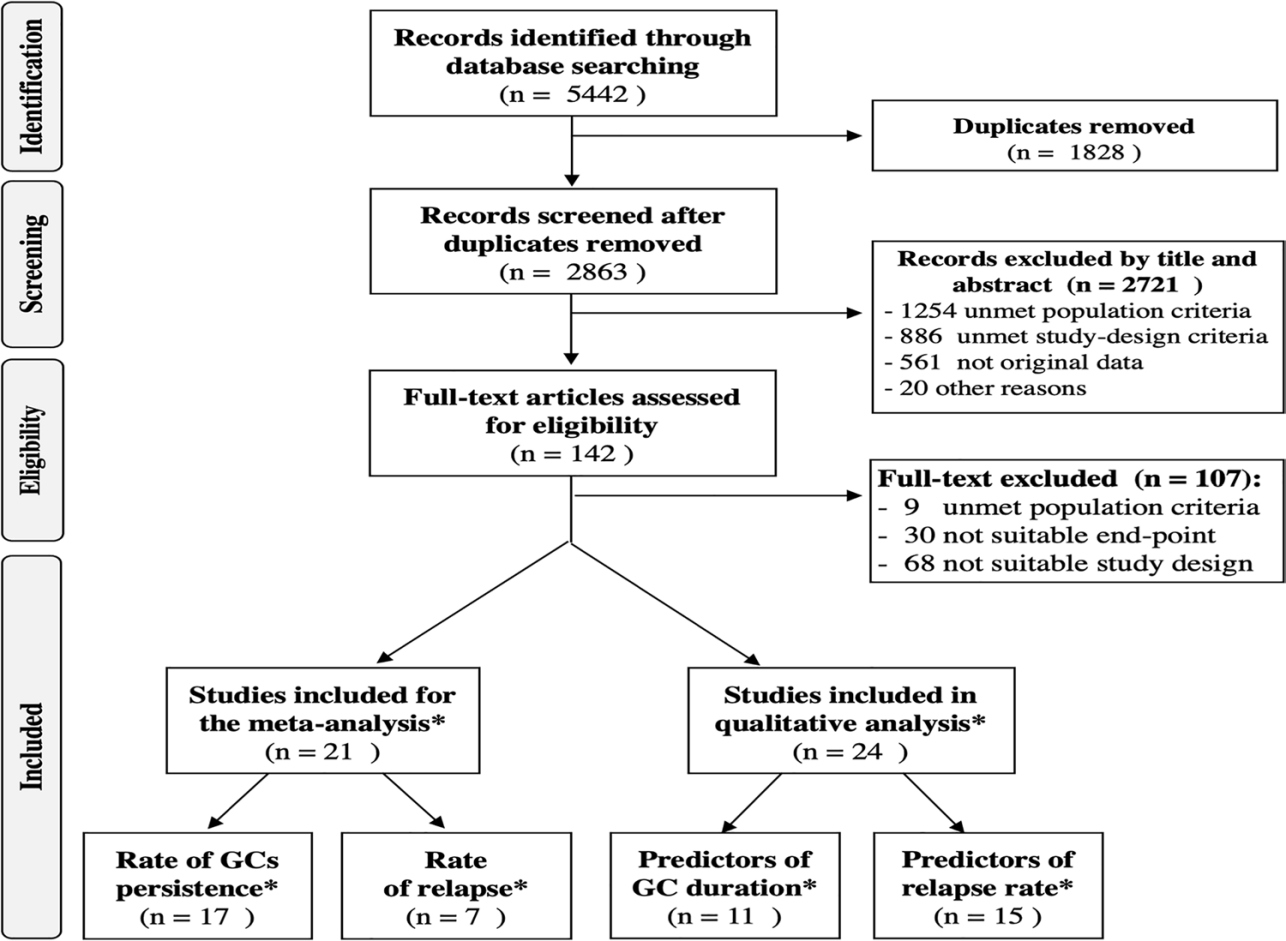
Flow chart diagram representing results of the process for selection of the retrieved studies. * Several studies were eligible both for quantitative and qualitative analysis and for more than one outcome

The results of the literature search and selection of articles are presented in Fig. 1. The electronic search strategy identified 5442 articles. After excluding duplicate articles, 2863 were selected. After screening by titles and abstracts, 142 were retrieved for a full review. Ultimately, 21 articles were selected for meta-analysis [4, 15–34], and 24 for qualitative analysis [15, 17, 20–23, 25, 26, 28, 29, 33–46]. All the selected studies showed a moderate to high quality, as assessed by the adapted JBI assessment method. Several studies were eligible both for quantitative and qualitative analyses and for more than one outcome.

### Long-term treatment with GCs in PMR

Details on the studies selected for meta-analysis of data on the persistence of GC treatment are reported in Table 1.

**Table 1:** Characteristics of the studies selected for the meta-analysis on the proportion of patients with polymyalgia rheumatica still on glucocorticoids at different time points

Author, year [ref]	Assessment time (yrs)	Patients (N)	Type of study	Recruitment period	Classification of PMR	PDN start dose(mg/day)
Aoki A, 2020 [15]	2	64	R	2011–2020	ACR/EULAR and Bird’s criteria	M 13.5
Mørk C, 2020 [16]	1, 2	174, 173	R/P	2012–2017	Physician’s diagn	Me 15
Marsman DE, 2020 [17]	1, 2	441, 357	R	2008–2018	Physician’s diagn	Me 15
Muller S, 2019 [18]	1, 2	493, 437	P	2012–2014	Physician’s diagn	M 15.6
van Sleen Y, 2019 [19]	2	19	P	2010–2018	Physician’s diagn	Me 15
Giollo A, 2019 [20]	2	205	P	na– > 2017	ACR/EULAR	NA
Albrecht K, 2018 [21]	1, 2	526, 315	P	2007–2014	Physician’s diagn	Me 7.5
Shbeeb I, 2018 [22]	1, 2, 5	334, 302, 201	R	200–2014	ACR/EULAR	M 16.9
Miceli MC, 2017 [23]	1	66	P	na	ACR/EULAR	0.2 mg/kg/day
Mackie SL, 2015 [24]	1	21	R	na	Bird’s	15 per protocol
Mazzantini M, 2012 [4]	2	222	R	na – > 2009	Bird’s	M 15
Mackie SL, 2010 [25]	5	164	R	1989–2000	Bird’s	29% > 15 mg/day
Cimmino M, 2008 [26]	5	57	Obs.ext. of RCT	1998–1999	Chuang’s	NA
Kremers H, 2007 [27]	5	364	R	1970–1999	Physician’s diagn	Me 15
Myklebust G, 2001 [28]	1, 2	217, 217	P	1987–1994	Bird’s criteria	Me 15
Weyand CM, 1999 [29]	1	27	P	1993–1996	Descriptive	20 per protocol
Ayoub WT, 1985 [30]	1, 2	75, 75	R	1975–1982	Descriptive	M 22.8

*Yrs*, years; *N*, number; *P*, prospective; *R*, retrospective; *NA*, data not available; *Rheum*, rheumatology; *Diagn*, diagnosis; ACR, American College of Rheumatology; EULAR, European League Against Rheumatisms; M, mean; Me, median

**Fig. 2:**
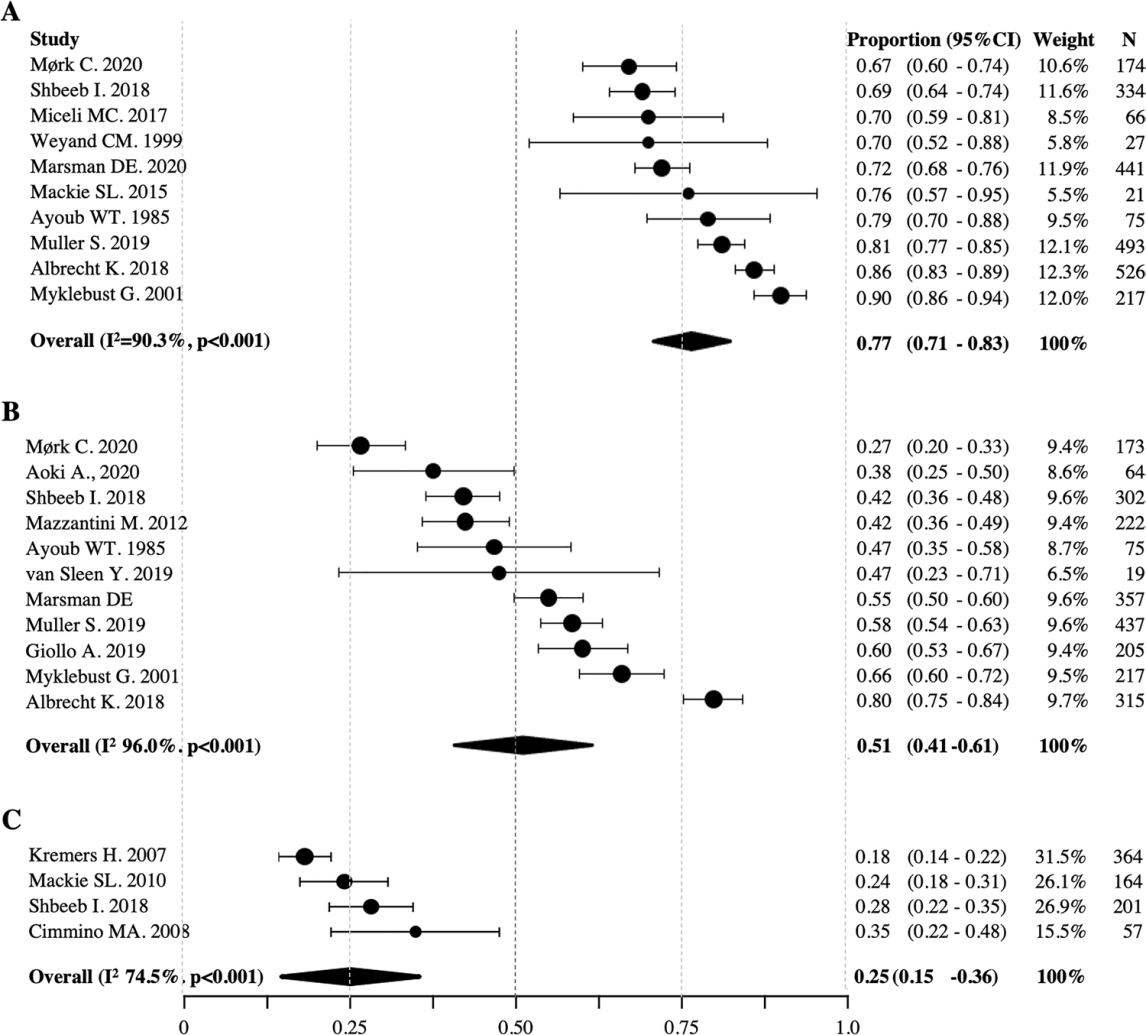
Forest plot of pooled proportion of patients still on glucocorticoids at (**A**) 1 year, (**B**) 2 years, and (**C**) 5 years from treatment initiation. 95%CI, confidence interval. N, number of patients recruited in each centre. I^2^, test for heterogeneity

Pooled data from 10 studies (population size: 2374) [16–18, 21–24, 28–30] showed that the proportion of patients still taking GCs at 1 year was 77% (95%CI 71–83%, I^2^ 90.3%) (Fig. 2a). Pooled data from 11 studies (population: 2260) [4, 15–22, 28, 30] showed that the proportion of patients still on GCs at 2 years was 51% (95%CI 41–61%, I^2^ 96.8%) (Fig. 2b). Pooled data from 4 studies (population: 786) [22, 25–27] showed that the proportion of patients still on GCs at 5 years was 25% (95CI% 15–36%, I^2^ 74.5%) (Fig. 2c). Only one study [21] reported the rate of GC persistence at 3 years (75%), and none at 4 years. All meta-analyses were characterized by high heterogeneity (I^2^ > 40%), but not significant publication bias (Egger’s test > 0.1).

The subgroup analysis discriminating cohorts predominantly recruited before and after the publication of the BSR recommendations in 2010 showed that the rates of persistence of GCs were respectively 79% (95%CI 70–89%, I^2^ 90.1%) [21, 22, 24, 28–30] vs. 73% (95%CI 63–83%, I^2^ 83.9%) [16–18, 23] at 1 year; and 55% (95%CI 35–76%, I^2^ 97.1%) [4, 21, 22, 28, 30] vs. 48% (95%CI 33–62%, I^2^ 93.5%) [15–20] at 2 years.

The subgroup analysis based on the retrospective or prospective nature of the studies showed that the retention rates of GCs were respectively 75% (95%CI 68–82%, I^2^ 79.4%) [17, 18, 22, 24, 30, 47] vs. 78% (95%CI 65 to 92%, I^2^ 90,3%) [16, 21, 23, 28, 29] at 1 year; and 49% (95%CI 40–58%, I2 85.6%) [4, 15, 17, 18, 20, 22, 30] vs. 55% (85%CI 18–72%, I^2^ 98.2%) [16, 19, 21, 28] at 2 years.

Finally, the meta-regression analysis did not demonstrate a significant effect of publication year on the variance of the estimated pooled GC retention at different time points (p = 0.356 at 1 year; p = 0.437 at 2 years). The influence of MTX use on the GC persistence rate was not investigated by meta-regression analysis because of the low number of studies reporting details on the proportion of patients on such treatment.

Subgroup analyses and meta-regression analysis were not performed for the persistence of GC treatment at 5 years, because of the inadequate number of studies.

### Rate of relapse in PMR

Details on studies selected for meta-analysis on the relapses rate are reported in Table 2.

**Table 2:** Characteristics of the studies selected for the meta-analysis on the relapse rate

Author, year	Assessment times (yrs)	Patients (N)	Type of study	Recruitment period	classification Criteria	Criteria for relapse
Mørk C, 2020 [16]	1	174	R / P	2012–2017	Physician’s diagn	C, L
Ayano M, 2020 [31]	1	32	R	2011–2017	Bird’s	C, L, T
Do JG, 2018 [32]	1	34	R	2009–2017	ACR/EULAR	C, L
Mackie SL, 2015 [24]	1	21	R	NA	Bird’s	NS
Lee JH, 2013 [33]	1	39	R	NA	Bird’s	C, L
Macchioni 2009 [34]	1	57	P	NA	Descriptive	C, L
Weyand CM, 1999 [29]	1	27	P	1993–1996	Descriptive	NS

*Yrs*, years. *N*, number. *P*, prospective. *R*, retrospective. *NA*, data not available. *Rheum*, rheumatology. *Diagn*., diagnosis. *ACR*, American College of Rheumatology. *EULAR*, European League Against Rheumatisms. *C*; clinical (reappearance or worsening of symptoms). *L*, laboratory (increased APRs). *T*, therapeutic (required increase of therapy)

**Fig. 3:**
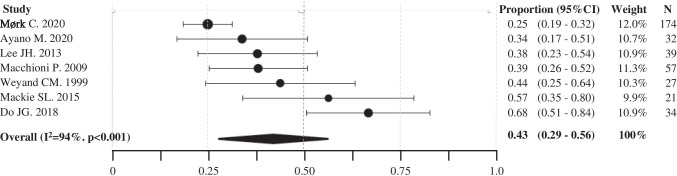
Forest plot of pooled proportion of patients experiencing at least 1 relapse at 1 year from treatment initiation. 95%CI, confidence interval. N, number of patients recruited in each centre. I^2^, test for heterogeneity

Pooled data from 7 studies (total patients 384) [16, 24, 29, 31–34] showed that 43% (95%CI 29–56%, I^2^ 94%, Egger’s tests p 0.031) of patients experienced at least one relapse after 1 year from treatment initiation (Fig. 3). One study on 173 PMR patients evaluated the relapse rate at 2 years (22.5%) [16]; two studies, respectively 53 [26] and 169 [24] patients, reported the relapse rate at 5 years (49% and 38%, respectively). None of the reviewed studies provided data at 3 and 4 years.

The publication years, the MTX use, and the different definition of relapses were not investigated by meta-regression analysis because of the insufficient number of studies.

Several studies reported the prevalence of patients who had at least one flare during an extremely variable follow-up period, making the outcome not suitable for meta-analysis. From these studies, those selected for qualitative analysis [25, 33, 34, 36–38, 40, 41, 43–45, 45] reported a proportion of patients experiencing at least one relapse during the entire follow-up (1 month–6 years) ranging between 22 and 67%. Three studies [37][37] [34] reported that 8–19% of patients experienced more than one relapse. When the time to relapse was reported, it was within the 2 years in most of the studies. Finally, recurrences, as defined by the reappearance of disease manifestation after a variable period of GC-free remission, were evaluated separately; their rate widely ranges between 5 and 37% (time from GCs stopping to recurrence up to 44 months) [38][36, 43, 49].

### Predictors of prolonged GC treatment and relapse

Details of the studies selected for qualitative analysis on predictors of GC treatment duration and occurrence of disease relapses are reported in Tables 3 and 4.

**Table 3:** Details of the studies investigating the potential predictors of long-term treatment with glucocorticoids (GCs)

Author, year	Patients (N)	Type of Study	Recruitment period	Classification criteria	Potential predictors of long-term GC treatment
Hattori K, 2020 [35]	50	R	2010–2017	Bird’s ACR/EULAR	**Normalization of *CRP*** at 1 month associated with higher likelihood of achievement of GC-free remission (OR = 5.83). No association was recorded with age and sex
Aoki A, 2020 [15]	93	R	2011–2020	Bird’s ACR/EULAR	**Relapse till 6 months** associated with long-term GC therapy (OR 6.40). No association was demonstrated with age, sex, APR, GC starting dose
Marsman DE, 2020 [17]	454	R	2008–2018	Physician’s diagn	**Normal APR** had shorter median time to GC-free remission (552 vs. 693 days). However, when the GC-retention rate at 1 and 2 years were evaluated, no significant differences were identified. Analysis focused on APR; thus, other candidate predictors were not assessed
Giollo A, 2019 [20]	385	R	< 2017	ACR/EULAR	**Older age** (adjHR, 1.02)**, peripheral involvement** (adjHR 1.38), **higher CRP** (adjHR 1.29**)**, **higher initial dosage of GC** (adjHR 0.96), **higher hemoglobin** (adjHR 0.86), **osteoporosis (adjHR0.75)**, and the use **of amino bisphosphonates** (adjHR0.65) associated with persistence in GC therapy. A trend to significant association was recorded with relapses. No association with sex and MTX or other DMARDs was recorded
Albrecht K, 2018 [21]	172	P	2007–2014	Physician's diagn	**Baseline MTX** (OR 2.03) **GCs > 10 mg/day** (OR 1.65), **higher disease activity** (OR 1.12) (median 0.6 years DD), and **female sex** (OR 1.63 [1.09–2.43]) were predictive for GC therapy at ≥ 3 years. No association was found with age and APR
Shbeeb I, 2018 [22]	359	R	200–2014	ACR/EULAR	**Initial dose of GC was not** associated with time to permanent discontinuation (HR 1.06 per 5 mg/day increase, 95% CI 0.96–1.18). Other possible predictors were not assessed
Miceli MC, 2017 [23]	66	P	na	ACR/EULAR	N of GC-free patients at 12 months was comparable among patients with or without **musculoskeletal ultrasonography (MSUS)** inflammatory findings at the baseline [14 (30.4%) in MSUS-positive vs 6 (30.0%) in MSUS-negative
Mackie SL, 2010 [25]	22	R	1989–2000	Bird’s	A higher plasma viscosity increases the risk of prolonged steroid therapy and late GCA. Starting patients on > 15 mg prednisolone is associated with a prolonged steroid duration. Age and sex did not associate with risk of prolonged GC duration
Cimmino MA, 2008 [26]	57	Obs. Ext. of RCT	1998–1999	Chuang’s	No GC-sparing effect of MTX was demonstrated. Other DMARDs were not assessed. Age, sex and APR did not associate with GC treatment duration
Myklebust G, 2001 [28]	217	P	1987–1994	Bird’s	Higher **mean maintenance GC dose in 1st yr** (6.1 vs. 4.8 mg/day of PDN), higher mean pretreatment ESR (73 vs. 60 mm/h) lower hemoglobin (12.3 vs. 12.9 g/dL). No significant association with initial GC dosage and APR
Weyand CM, 1999 [29]	27	P	1993–1996, > 1 yr	Physician’s diagn	**ESR** and non-responsiveness of interleukin 6 to steroid therapy are helpful in dividing patients into subsets with different treatment requirements

*P*, prospective; *R*, retrospective; *Obs.ext. of RCT*, observational extension of a randomized clinical trial; *Mths*, months, yrs; *Yrs*, years; *Rheum*, rheumatology; *APR*, acute-phase reactants; *CRP*, C-reactive protein; *ESR*, erythrocyte sedimentation rate; *OR*, odds ratio; *HR*, hazard ratio; *adjHR*, adjusted HR; *NA*, not available; *MTX*, methotrexate; *ACR*, American College of Rheumatology; *EULAR*, European League Against Rheumatisms

**Table 4:** Details of the studies investigating the potential predictors of relapses

Author, year	Patients (N)	Type of study	Recruitment period	Classification criteria	Relapse criteria	Relapse rate	Potential predictors of relapses
Lee JH, 2013 [33]	39	R	na	Bird's	C, L	15 (38%)	**Initial CRP** > 2.5 mg/dl (OR 6.3) and the use of **hydroxychloroquine** (OR 6.798). Female gender (OR 10.683, p = 0.052). MTX and other DMARDs did not associated with occurrence of relapse
Macchioni P, 2009 [34]	57	P	na	na	C, L, T	22 (39%)	**Positive PD** signal at diagnosis (63.6 vs 15.4%)
de la Torre ML, 2020 [36]	86	P	2017		C.L	40 (47%) 15 (17%)	**MTX** in PMR patients who already had a relapse reduced the number of future relapses and decreased the time to achieve remission
Fukui S, 2016 [37]	115	P	2004–2013	ACR/EULAR	C, L, T	29 (23%)	**Female gender** (OR 2.73) and creatinine > 50 μmol/L (OR, 2.48) associated with occurrence of relapses. No association was reported between occurrence of relapse and age, APR, GC initial dose, speed of GC tapering and peripheral arthritis
Kimura M, 2012 [38]	123	R	2000–2009	Hunder’s	C, L	29 (23%); 7 (5.7%)	No significant difference with RS3PE
Mackie SL, 2010 [24]	169	R	1989–2000	Bird’s	C, T	83 (49.1%)	No association was recorded between occurrence of relapses and age, sex, APR and GC initial does
Boiardi L, 2006 [39]	112	P	1993–1997	ns	C, L	49 (43.7%)	Persistently **elevated IL- 6 levels**, but not the CC genotype
Cimmino MA, 2008 [26]	57	Obs. Ext. of RCT	1998–1999	Chuang’s	C. L.T	20 (35%)	Flare- ups of PMR were seen in 8/26 (30.8%) MTX-treated patients in comparison with 12/27 (44.4%) controls
Salvarani C, 2005 [40]	94	P	1994–1997	Descriptive	C, L, T	47 (50.0%)	Persistently elevated levels of **CRP** (RR ranging between 2 and 5) and persistently elevated levels of **IL-6** (RR 4–13) associated with occurrence of relapses. Age, sex and GC initial dose and peripheral arthritis did not predict relapses
Kremers HM, 2005 [41]	284	R	1970–1999	Descriptive	C, T	55%	Higher ESR (HR 1.14); higher **initial GCs dose** (HR 1.07); **fast tapering** of GCs (HR 4.27), medium CS tapering (HR 2.19). Age, sex and peripheral arthritis did not associate with relapses
Martínez-Taboda VM, 2004 [42]	54	R	na	Descriptive	C, T	18 (33.3%)	Increased expression of the HLA-DRB1*09 allele (5.6% vs 0%)
Gonzalez-Gay MA, 2002 [43]	86	R	na	Descriptive	C, L*, T	18 (20.9%) 3 (3.5%)	No differences in CRH-A or B alleles and genotypes
Amoli MM, 2002 [44]	72	R	na	Descriptive	C, T	18 (22%)	HLA-DRB1*0401 and the ICAM-1 codon 241 GG homozygosity
Boiardi L, 2000 [45]	92	P	1992–1996	Descriptive	C, L, T	40 (44%)	IL-1A (+ 4845), IL-B (-511), IL-B (+ 3954), IL-1RN Intron 2 VNTR and TNFA (-308) no associated with the disease severity
Salvarani C, 2000 [46]	88	P	1992–1996	Descriptive	C, L, T	37 (42%)	No association with CCR5∆32

*P*, prospective; *R*, retrospective; *Obs.ext. of RCT*, observational extension of a randomized clinical trial; *Mths*, months, yrs; *Yrs*, years; *Rheum*, rheumatology; *APR*, acute-phase reactants; *CRP*, C-reactive protein; *ESR*, erythrocyte sedimentation rate; *OR*, odds ratio; *HR*, hazard ratio; *adjHR*, adjusted HR; *NA*, not available; *MTX*, methotrexate; *ACR*, American College of Rheumatology; *EULAR*, European League Against Rheumatisms

#### Age

Among 5 studies [15, 20, 25, 26, 35] investigating age as a potential predictor of treatment duration with CGs, only one [20] recorded a significant association between older age and longer exposure to GCs (adjHR, 1.02, 95%CI 1.01–1.04). Furthermore, age was associated with the relapse risk in none of 6 studies [25, 33, 36, 37, 40, 41], where such an outcome was assessed.

#### Gender

Only 1 study [21] of 6 [15, 20, 21, 25, 28, 35] identified female gender as a predictor of long-lasting therapy with GCs (OR 1.63, 95%CI 1.09–2.43 for treatment duration ≥ 3 years).

Similarly, females were significantly more likely to have at least one relapse (OR 2.73, 95%CI 1.16–6.41) in one study [37], but not in the other five [24, 33, 37, 40, 41].

#### Acute phase reactants

Higher acute phase reactants (APRs) have been investigated as predictors of treatment persistence in 8 studies [15, 17, 20, 26, 28, 29, 35, 47]. Giollo et al. [20] demonstrated that a higher baseline value of C-reactive protein (CRP) predicted a persistent treatment with GCs over a median follow-up of 30 months (adjHR 1.29, 95%CI 1.14–1.45). Accordingly, Marsman et al. [17] reported that patients with normal APRs at baseline had a shorter time to achievement of GC-free remission (552 vs 693 days); and Hattori et al. [35] reported that normalization of CRP at 1 month was associated with a high likelihood of achievement of GC-free remission (OR = 5.83, 95%CI 1.28–26.51). However, 4 studies [15, 21, 26, 28] failed to demonstrate a significant predictive value of APRs for treatment duration.

An increased likelihood for the occurrence of relapses was recorded for the value of CRP > 2.5 mg/dL (OR 6.30, 95%CI 1.03–38.67) in one study [33]. Furthermore, persistently elevated levels of CRP during the first months of treatment were associated with increased risk of relapse (RR ranging between 2 and 5) [40], and higher erythrocyte sedimentation rate value associated time to first relapse (HR 1.14, 95%CI 1.05–1.23 per 10 mm/h increase) [41]. Other 3 studies did not demonstrate a predictive role of higher values of APR for relapses [24, 36, 37].

#### Starting dose of GCs and speed of tapering

Regarding the starting GC dosage, Giollo et al. [20] reported that a higher initial dose of prednisone, or equivalents, was negatively associated with a persistent duration on treatment over a median follow-up of 38 months (HR 0.96, 95%CI 0.95–0.98). Conversely, Albreight et al. [21] reported that baseline GC dosage > 10 mg/day was associated with a treatment duration > 3 years (OR 1.65, 95%CI 1.07–2.55), and Mackie et al. [24] reported that patients with baseline GC dosage > 15 mg/days were less likely to stop steroids within 5 years (adjHR 0.56, 95%CI 0.34–0.91). Further 4 studies [15, 22, 28] did not identify a significant correlation between the initial dose of GCs and overall treatment duration.

Initial dose of GCs was associated to a higher risk of relapse (HR 1.07, 95%CI 1.02–1.13) in 1 [41] out of 5 [25, 33, 37, 40, 41] studies and speed of tapering in 1 [41] of 3 [33, 37, 41] studies.

#### Peripheral arthritis

Peripheral arthritis at baseline was associated with longer treatment with GCs in one study (adjHR 1.38, 95%CI 1.05–1.83) [20]; whereas the significant association was denied in other 2 articles [15, 23]. Furthermore, peripheral arthritis was not associated with the occurrence of relapse in 4 studies [33, 37, 40, 41].

#### Methotrexate and other potential steroid-sparing agents

A significant association between using MTX (unspecified dose) from baseline and GC treatment duration ≥ 3 years (OR 2.03, 95%CI 1.27–3.24) was recorded in one prospective observational study [21]. Conversely, no association between MTX (10 mg weekly) since baseline and shorter GC treatment duration was recorded in an observational 5-year extension of a randomized controlled trial (RCT) [26].

No protective effect against the occurrence of relapses was identified for using MTX since diagnosis (unspecified dosage) in an observational study [33], and in the RCT extension study (10 mg weekly). [26] On the other hand, using MTX 15 mg weekly in patients who already had a relapse was recorded to reduce the number of future relapses and the time to achieve remission in a prospective study [36].

Hydroxychloroquine use from baseline associated with lower likelihood of relapse (OR 6.798, 95%CI 1.145–40.372) in a prospective observational study [33]; but when it was assessed along other disease-modifying anti-rheumatic drugs (DMARDs), including MTX (7.5–20 mg/week), no steroid-spearing effect was recorded [20]. No further data were found for other conventional DMARDs as steroid-sparing agent or preventive treatment for disease relapse.

## Discussion

Thanks to the prompt suppression of its disabling symptoms by low-moderate doses of GCs, PMR is commonly considered a benign disorder. However, when PMR patients are carefully followed-up over the years, and long-term outcomes are taken into account, significant concerns arise regarding the duration of treatment and the occurrence of relapses. In this study, we attempted to summarize current evidence on magnitude and predictors of prolonged GC treatment and the occurrence of relapses in a real-life setting.

The pooled data from observational studies showed a high GC persistence rate over time, pointing out a significant gap between what is recommended and what happens in daily clinical practice. Indeed, 77%, 51%, and 25% of PMR patients were still on GCs after respectively 1, 2, and 5 years from treatment initiation. The publication of the first international recommendations [5] for PMR management about 10 years ago does not seem to have significantly affected the overall rate of steroid treatment prolonged for more than 1 and 2 years.

Although disease relapses were expected to be the main determinants of persistence in PMR treatment, few studies have formally assessed the association between relapses and long-term GC therapy. In our review, the proportion of patients experiencing at least one relapse was 40% at 1 year (by meta-analysis) and 20–67% on a variable follow-up duration ranging from 1 month to 6 years (qualitative analysis). Albeit remarkable, such values suggest that relapses can only partially explain the high persistence of steroid treatment in PMR; thus, other factors are involved, including a proportion of patients not achieving complete remission with GCs, a general reluctance of clinicians to early discontinue steroids, or an incomplete awareness of the potential consequence of long-term exposure to GCs.

Several studies have attempted to identify potential predictors of prolonged treatment with GCs and relapse, but with inconsistent results. Female gender, APR levels, peripheral arthritis, starting GC dosage, and tapering speed were the most frequently investigated factors. Still, none of them demonstrated a univocal correlation with the duration of GC treatment nor with the occurrence of relapses. Poor results are also derived from pharmacogenomics. Such conflicting data are consistent with the findings of the systematic literature review [50] informing the 2015 ACR/EULAR recommendations, where the authors found in low- to moderate-quality studies that baseline factors associated with a higher relapse rate and prolonged therapy were female sex, elevated ESR (> 40 mm/1st hour), and peripheral inflammatory arthritis. However, they also reported that some equally low- to moderate-quality studies failed to demonstrate such association. In the final document [6], on the one hand, authors stated that the role of risk factors for relapse and long-term therapy is not clear yet; on the other hand, authors recommend considering the early introduction of MTX in patients at high risk for relapse or prolonged treatment, as well as in cases with comorbidities.

Only a few retrieved studies evaluated the potential role of early treatment with MTX in reducing the duration of GC exposure and the risk of flare-ups in PMR, reaching to inconsistent results. Thus, data from the observational studies do not help in solving the uncertainty derived from RCTs. Indeed, on the one hand, there is evidence from some clinical trials indicating a benefit of MTX on relapse rate, discontinuation of GC, and cumulative GC dose [51] [52]; but, on the other hand, there is other evidence from different studies indicating no effect of MTX on the same outcomes [53][53]. Even though a higher quality of evidence was identified in studies reporting the efficacy of MTX, a stronger recommendation for the use of MTX is not supported by the experts because the total number of patients investigated RCTs (n = 194), the not univocal results, and the lack of demonstration of a reduction in GC‐related adverse events [6]. Promising data emerged from some clinical series on the use of interleukin-6 (IL-6) inhibitors in GC-resistant PMR patients [55]; however, more evidence are needed from specific clinical trials and real-life experience gained in wider cohorts [56].

Overall, both data from observational studies and RCTs point out that the availability of effective and safety GC sparing agent is still an unmet need in PMR. In this regard, it is suggested that future research aimed to develop targeted therapies for these patients should take in account the recognition of a prevalent (auto)inflammatory, rather than autoimmune, nature of PMR [57].

This study has some strengths. First, the applied search strategy, even requesting a greater effort in the selection process, ensured a higher comprehensiveness of the results by minimizing the risk of missing eligible studies. Second, in accordance with the objective of the study, the sole selection of observational studies with the exclusion of clinical trials [51, 52, 54, 58–61], characterized by scheduled tapering regimens, allowed to have a more reliable representation of the clinical practice in a real-life setting. Third, the exclusion of studies recruiting patients with concomitant GCA and patients whose diagnosis was changed to elderly onset rheumatoid arthritis prevented the significant bias of an incorrect attribution to PMR of long-term requirement of GCs and occurrence of relapses.

The major limitation of this work may be the high heterogeneity of selected studies. However, because of the descriptive nature of the investigated outcomes, such heterogenicity does not affect the result’s validity and overall clinical significance. Still, it is representative of the variable approaches in the management of PMR in a real-life setting. Furthermore, although this review was conducted with a rigorous methodology according to the PRISMA statements, the lack of registration into the PROSPERO International prospective register of systematic reviews may represent a limitation.

In summary, although the international recommendations and the wide consensus that GCs should be used as shorter as possible (ideally by 1–2 years), a high rate of prolonged steroid treatment is still recorded in the management of PMR. The recorded relapse rate, even remarkable, can only partially explain the requirement of long-term GC treatment, suggesting that other and not yet identified factors may be involved, including clinicians’ general reluctance to early discontinue steroids or incomplete awareness of the potential consequence of prolonged exposure to GCs. Moreover, the lack of validating predictors for long-term GC treatment and relapse prevents the personalization of the patients’ management. The results of this study suggest that additional research is needed to understand the reasons for prolonged treatment, especially in patients who have not relapsed, enhance the adherence to the recommendations, develop evidence-based strategies for GCs tapering, clarify the role of MTX and other potential steroid-sparing agents, and identify biomarkers for an individualized disease management.

## Data Availability

The dataset generated during the literature review is not publicly available, but it is available from the corresponding author on reasonable request.
